# Obituary

**Published:** 1883-01

**Authors:** 


### Obituary.

The Death of Sir Thomas Watson, the Nestor of British physicians, at the advanced age of ninety, removes from the medical ranks the last of what may be accurately called the old school of physicians. Sir Thomas is known among us chiefly by his "Practice of Physic," a work that forms the first reading "in practice" of the majority of students. Sir Thomas described with an accuracy and insight that left nothing to be desired. His book (almost his only literary work) was essentially for the practitioner, and even to-day we turn to it for opinions and pictures of disease that have not, nay, cannot be excelled.

Sir Thomas was graduated from Cambridge University in 1815, and was knighted in 1866. At the time of his death he was physician-in-ordinary to the Queen.

H. S. McCall, M. D., M. D. S., died at his residence in Binghamton, N. Y., December 10. Dr. McCall was born in Franklin, Delaware County, N. Y., June 24, 1824. He graduated from the Woodstock Medical College and began the practice of medicine in his native town, subsequently removing to Batavia, N. Y. Forsaking general medicine, he entered the office of the late Dr. Fellows, at Albion, and began the study of dentistry. In 1855 he removed to Binghamton and opened an office, soon becoming widely known as a skilful and conscientious operator. He was prominently connected with the New York State and the Sixth District Dental Societies, where his unassuming manner and genial temperament won him hosts of friends, who will sincerely mourn his death.

Death of M. Davaine.—In a recent number of *Le Progres Medical* we find a note announcing the decease of M. Davaine, at the age of seventy years. Davaine's contributions to helminthology are well known, and the journal alluded to credits his writings with having played a leading part in clearing up the question of the anthrax group of diseases in man and in the lower animals.

